# Supplementary material for: Causal Relationship between Aging and Anorexia Nervosa: A White-Matter-Microstructure-Mediated Mendelian Randomization Analysis
Source: Biomedicines. 2024 Aug 16;12(8):1874. doi: 10.3390/biomedicines12081874 (PMC11351342; doi:10.3390/biomedicines12081874)
Supplement: Supplementary file 1 [file biomedicines-12-01874-s001.zip › Supplementary Figure/Figure S8/Leaveoneout plot of MR analysis for TL to ubm-b-1764.pdf]

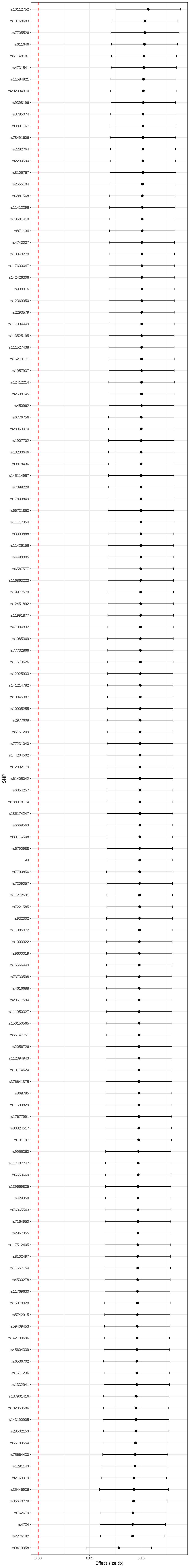

rs10112752
rs10768683
rs7705526
rs611646
rs61748181
rs4731541
rs11584821
rs202034370
rs9398196
rs3785074
rs3891167
rs78491606
rs2282764
rs2230590
rs8105767
rs2555104
rs6881568
rs11412296
rs73581419
rs871134
rs4743037
rs10840270
rs117630647
rs142426306
rs939916
rs12369950
rs2293579
rs117034449
rs113525195
rs111527438
rs76219171
rs1957937
rs12412214
rs2538745
rs450962
rs6776756
rs28363070
rs1907702
rs13230646
rs9878436
rs145114957
rs7099229
rs17803849
rs66731853
rs11117354
rs3093888
rs11426156
rs4498805
rs6587577
rs116863223
rs79977579
rs12451892
rs11991877
rs41304832
rs1985369
rs77732866
rs11579626
rs12925933
rs141214782
rs10845387
rs10905255
rs2977608
rs6751209
rs77231040
rs144204502
rs12932179
rs61405042
rs6054257
rs188918174
rs185174247
rs6669563
rs80116508
rs6790988
A8
rs7780856
rs7209057
rs11212631
rs7221585
rs932002
rs11085072
rs1003322
rs9600019
rs76666449
rs73730598
rs4616688
rs28577594
rs111950327
rs150150565
rs55747751
rs2056726
rs112394943
rs10774624
rs376641875
rs869785
rs11699829
rs17677991
rs80324517
rs131797
rs9955360
rs117407747
rs6659669
rs139669835
rs429358
rs76065543
rs7164950
rs2967355
rs117512405
rs8102497
rs11557154
rs4530278
rs11769630
rs16978028
rs5742915
rs59409453
rs142730696
rs45604339
rs6536702
rs1611236
rs1332941
rs137901416
rs182059586
rs143190905
rs28502153
rs56799554
rs75664430
rs1291143
rs2763979
rs35446936
rs35640778
rs762679
rs4724
rs2276182
rs9419958
SNP
0.00
0.05
0.10
Effect size (b)
